# Key Factors for Improving Rigor and Reproducibility: Guidelines, Peer Reviews, and Journal Technical Reviews

**DOI:** 10.3389/fcvm.2022.856102

**Published:** 2022-03-09

**Authors:** Hong S. Lu, Alan Daugherty

**Affiliations:** ^1^Saha Cardiovascular Research Center, University of Kentucky, Lexington, KY, United States; ^2^Saha Aortic Center, University of Kentucky, Lexington, KY, United States; ^3^Department of Physiology, University of Kentucky, Lexington, KY, United States

**Keywords:** technical review, statistical analysis, replicates, animals, cardiovascular medicine, vascular biology

## Abstract

To respond to the NIH's policy for rigor and reproducibility in preclinical research, many journals have implemented guidelines and checklists to guide authors in improving the rigor and reproducibility of their research. Transparency in developing detailed prospective experimental designs and providing raw data are essential premises of rigor and reproducibility. Standard peer reviews and journal-specific technical and statistical reviews are critical factors for enhancing rigor and reproducibility. This brief review also shares some experience from *Arteriosclerosis, Thrombosis, and Vascular Biology*, an American Heart Association journal, that has implemented several mechanisms to enhance rigor and reproducibility for preclinical research.

## Introduction

In the past 50 years, the number of scientific publications has grown from ~300 K per year to above 1,000 K every year. During this interval publication, retractions have also increased rapidly. Retractions cover a spectrum of causes including errors, misconduct, fabrication, or fraud. These are well-publicized examples that hinder reproductivity. However, a broader and more insidious problem is inability to reproduce. One illustration is a survey published in *Nature* that found only six (11%) of 53 selected publications from academic laboratories being reproduced by a pharmaceutical company, raising serious concerns on rigor and reproducibility (1). The survey concluded that the studies they were able to reproduce were characterized by authors being attentive to controls, reagents, investigator bias, and describing complete datasets. In contrast, for results that could not be reproduced, limited methodological information was provided, and experiments were not rigorously performed or selected with bias.

In 2012, the United States National Institute of Neurological Disorders and Stroke convened a meeting to develop recommendations for improving preclinical research (2). This initial call for transparent reporting prompted subsequent discussion and implementation of rigorous experimental design. In 2014, the National Institutes of Health (NIH) held a joint workshop with the Nature Publishing Group and Science to discuss issues related to rigor and reproducibility of research articles. Subsequently, in 2016, the NIH announced a Rigor and Reproducibility policy and guidelines for scientific research, including transparency in reporting detailed methods and rigorous statistical analyses. The NIH has also provided a resource chart (https://grants.nih.gov/grants/RigorandReproducibilityChart508.pdf) to emphasize four areas of focus for grant applications:

1. Scientific premise (the rigor of previous experimental design and data analysis).
2. Scientific rigor for experimental design of the proposed research.
3. Biological variables (particularly sex).
4. Authentication of key biological and/or chemical resources.

In response to the NIH's policy and guidelines, many journals have implemented multiple guidelines and certain checklists for manuscript submission. *Arteriosclerosis, Thrombosis, and Vascular Biology* (*ATVB*), an American Heart Association (AHA) journal, is a well-recognized cardiovascular research journal that receives ~1,500 manuscripts every year. We will use *ATVB* as an example when we discuss some parts, especially the technical review section, in this mini review.

## Implementation of Principles and Guidelines Is Necessary to Improve Transparency, Rigor, and Reproducibility

Multiple guidelines have been launched for appropriate study design and data analysis as well as transparent reporting of animal research (Table 1). One important guideline is the Transparency and Openness Promotion (TOP) guidelines. The TOP guidelines provide a category of “not implemented” for journals that have no declared policy for sharing experimental details. For journals that require a declaration, the guidelines provide three levels for data transparency:

1. Level 1 requires that authors disclose whether data are available and, if so, where to access them.
2. Level 2 requires that data must be posted to a trusted repository, while exceptions must be identified at manuscript submission.
3. Level 3 requires that reported analyses must be reproduced independently by a third party, in addition to the request in Level 2.

**Table 1:** Selected major guidelines for transparent reporting.

**Abbreviation**	**Full name**	**Scope**	**Reference or link**
ARRIVE	Animal research: reporting of *in vivo* experiments	Checklist of recommendations to improve the reporting of animal research	References (3, 4)
MIAME	Minimum information about a microarray experiment	Checklist for microarray studies	https://www.ncbi.nlm.nih.gov/geo/info/MIAME.html
MINSEQE	Minimum information about a next-generation sequencing experiment	Checklist for sequencing studies	https://www.ncbi.nlm.nih.gov/geo/info/MIAME.html
NIH	National Institutes of Health	Policies and guidelines for preclinical and clinical research	https://grants.nih.gov/policy/reproducibility/index.htm
TOP	Transparency and openness promotion	Journal publishing standards for transparency and data sharing	https://www.cos.io/initiatives/top-guidelines

Most scientific journals, including *ATVB*, require the transparency of Level 1, to state the availability of data in manuscripts. More recently, many journals are taking efforts toward Level 2, especially for large datasets such as bulk RNA sequencing data, single cell transcriptomics data, and proteomics data. Gene Expression Omnibus (GEO) and European Nucleotide Archive (ENA) have been used as appropriate and trusted repositories for nucleotide transcriptomics, and the ProteomeXchange Consortium developed through PRIDE is for mass-spectrometry-based proteomics repository. bioRxiv, hosted by the Cold Spring Harbor Laboratory, is a popular open-access preprint repository for biological sciences. The NIH and some journals encourage authors to post their manuscripts with detailed methods and raw data for preclinical research in bioRxiv prior to submission. To facilitate manuscript submission to a journal, direct submissions are available from bioRxiv to an increasing number of journals. Attributed to the enhanced reputation of bioRxiv, medRxiv was launched in 2019 as an open-access preprint repository for clinical and populational research. Although bioRxiv and medRxiv post original research without peer review process, they are read and commented by more researchers who are interested in the Research Topics. The dialog established by this open forum system has been considered to be a more rigorous “review process” that offers opportunities for authors to correct apparent errors prior to its publishing in a journal.

For animal research, the Animal Research: Reporting of *in vivo* Experiments (ARRIVE) guidelines published in 2010 (3) has become a “gold” standard for many journals. Recently, the ARRIVE guidelines 2.0 (4) introduced ARRIVE Essential 10 as the basic minimum for manuscript submission. These include minimal recommendations for study design, prospective estimation of sample size, inclusion and exclusion criteria, randomization, blinding, outcome measures, statistical methods, detailed information of experimental animals, detailed experimental procedures, and result description. *ATVB* has been implementing Major Resources Table (https://www.ahajournals.org/atvb/manuscript-preparation) since 2017 that includes animal information (source, background strain, and sex), mouse breeding strategy, antibody information (source catalog number, batch number, and working concentration), and other necessary reagents. AHA journals are in the process of including ARRIVE Essential 10 in Major Resources Table.

The Advisory Committee to the Director (ACD) of the NIH working group on enhancing rigor, transparency, and translatability in animal research presented a report (https://www.acd.od.nih.gov/working-groups/eprar.html) in June 2021. The ACD working group recommends that the NIH should have detailed plans to identify gaps and opportunities and train animal researchers for appropriate experimental design and statistical analyses. The ACD working group also suggested an enhanced implementation of prospective registration for animal studies in a similar process as clinical trials. This process can be performed on an existing online animal study registry (https://www.animalstudyregistry.org/asr_web/index.action). It is likely that this will have increased emphasis in the near future.

Overall, Level 1 and Level 2 of the TOP guidelines for transparency are recommended for manuscript submission. ARRIVE guidelines, minimally Essential 10, should be implemented for designing animal studies prospectively and for reporting outcomes of animal research.

## Peer Review Is Needed for Publishing and Advancing Scientific Research

Peer review is a process in scientific journals to evaluate manuscripts of original research by experts in the same or similar field. Peer review critiques the significance and originality of a publication and provides suggestions to help authors improve quality, including identifying conceptual and technical errors that may impact data interpretation. Therefore, peer review is a fundamental process to assess quality, impact, and novelty of the science reported in a manuscript before it is published (5). Although peer review may be time-consuming, it could be mutually beneficial, because reviewers may be inspired by the manuscript to advance their own research. Despite its many benefits, some deficiencies of peer review have been noted.

In most journals, peer review is usually performed by two to three experts who are selected based on their expertise in the same subject matter as the submission. These are selected by an editor who preferably has knowledge of the subject area of the manuscript. The most common process is “single-blind” review; namely, the reviewers know the identity of the authors, but the authors are not informed of the identity of the reviewers. It is not infrequent that authors complain that peer review is neither fair nor constructive. In particular, if the authors' findings conflict with a reviewer's own research, it may lead to biased critiques. To overcome this potential deficiency of peer review, most journals suggest that authors provide names to exclude as a reviewer, mainly those who may have conflicts of interest. Many journals allow to exclusion of up to three potential reviewers by providing a reason for excluding the specific reviewers. Some journals provide the option post acceptance notification of reviewers' names and a link to their critiques. Some journals have implemented a double-blind process in which the reviewers are not provided with the identity of the authors, and *vice versa*. However, given the proclivity of citing previous publications, blinding of the authors' identity to the reviewers has challenges.

Another apparent deficiency of peer review is that it does not provide a systemic evaluation of plagiarism. Some journals have implemented some software tools to screen for plagiarism. The caveats of plagiarism software detection need to be considered. For example, it is common, and acceptable, to provide a description of methods that replicates the authors' own previous publications. Therefore, automated flagging of plagiarism, based on automated analysis, usually needs extensive manual verification.

Although the peer review process has flaws, it is a powerful tool to improve the quality of research manuscripts and deliver meaningful science to the scientific community. To minimize potentially biased comments, journal editors need to determine who are the most appropriate, qualified reviewers. It is not uncommon that the three reviewers have different opinions on a manuscript. In this case, editors also need to balance the comments based on the journal policies and their knowledge on the research. Therefore, journal editors are the driving factor for appropriate peer review process. For *ATVB*, the editors have developed a large, stable, and reliable reviewer network. They evaluate whether reviewers provide their comments on a timely manner with unbiased and constructive opinions. Also, for highly conflicted comments among the three reviewers, the handling editor can invite additional members of the editorial team to contribute to an online discussion forum prior to making a decision.

## Technical Review Provides Consistent Standard for Enhancing Rigor and Reproducibility

Historically, some journals, especially those publishing clinical and populational research, have statistical reviewers given the complexity relative to classical preclinical research (6). While concerns have been noted previously (7), statistical analysis-related impact has had more limited attention for preclinical research until the NIH launched its policies and guidelines. The present editors of *ATVB* have endeavored to improve manuscript quality and consistency. The initial steps included asking reviewers to fill a detailed checklist of their evaluation regarding the authentication of experimental design and statistical analysis. Unfortunately, this approach did not provide consistent evaluations. Indeed, the same question on authentication for a same manuscript could receive three different answers from the three reviewers. This is particularly critical when the question involves statistical analysis methods and appropriateness. Although the editors have continuously expanded the details of the technical guidelines for manuscript submission and peer review process, improvement in appropriate statistical analysis and methodology has been modest. In 2017, *ATVB* decided to appoint a technical review editor to provide a process for consistent evaluation of statistical analysis and methodology. This was applied to all original manuscripts receiving an editorial decision that permitted resubmission. This process has profoundly enhanced the transparency of methods, consistency and accuracy of statistical analysis methods, and rigor of experiments and biological replicates, and it considers sex as a biological variable. Significant improvement of publications in *ATVB* attributed to this technical review process has also prompted implementation of technical review editors in several other AHA journals.

The most common deficiencies of manuscripts submitted to *ATVB* that have been illuminated by the technical review process include:

1. Statistical analyses: conducting parametric tests without stating that datasets fit the constraints of variance and normality, and not conducting appropriate multigroup tests.
2. Small sample size such as 3 biological replicates per group.

To overcome the major issues noted, recently *ATVB* has included a new checklist for manuscript submission. This is also the checklist for technical review of *ATVB* (https://www.ahajournals.org/atvb/manuscript-preparation). Below, we summarize the checklist that focuses on appropriate statistical analysis:

1. Emphasize biological vs. technical replicates. Authors should clearly state how many independent samples per group (biological replicates) in each cohort were studied.
2. Data presentation should always show individual data for each figure. Additionally, it is suggested that data tested by parametric tests should be represented as mean and standard error of mean (SEM) or standard deviation (SD), whereas data tested by non-parametric tests should be represented as median with confidence limits.
3. Statistical analysis: data should be tested for both normality and equal variance as a pre-condition or justification for conducting parametric analysis.
4. For sample size *N* ≤ 5 per group, it is recommended either to perform a non-parametric statistical test or to increase sample size. This is because normal distribution and data homogeneity cannot be determined appropriately for small sample sizes.
5. Precise *P* values, rather than *P* < 0.05 or not significant (NS), should be provided.
6. Emphasize sex as a biological variable. Groups stratified by sex should be analyzed separately.

We also provide an excellent description of a statistical analysis that was published by Schneckmann et al. (8). This example states, “Sample size estimation was based on previous results in comparable studies, assuming 80% power at a significance level of 0.05. Specific details on how many independent biological samples or mice were included in an experiment or how many times experiments were repeated independently are given in the corresponding figure legends. Sample size is given in the figure legends and data are presented as mean±SEM. For continuous variables, normality and homogeneity of variance were assessed by Shapiro-Wilk and Brown-Forsythe tests, respectively. After confirming homogeneous variances and normality, two-group comparisons for means were performed by two-sided unpaired Student's *t* test. In case of inhomogeneous variances, Mann-Whitney *U* test was performed. Multi-group comparisons for means were performed by two-way ANOVA with Sidak multiple comparison test. *P* < 0.05 was considered statistically significant. Statistical analyses mentioned above were performed using “Graph Pad Prism version 9.1.0.” We hope that this can help authors understand how to describe and perform a statistical analysis appropriately.

## Perspective

Researchers publish medical scientific articles to promote academic career, advance medical science, and, ultimately, improve human health. One example that achieves these objectives is an article that was published in 2012 by Dr. Doudna and Dr. Charpentier in *Science* that has impacted science profoundly and the major premise has been reproduced by many researchers (9). Meanwhile, there is anecdotal acknowledgment that a substantial number of published studies cannot be reproduced by other researchers. We have discussed the key components for enhancing transparency, rigor, and reproducibility that rely on policies, guidelines, journal editors, peer review, and journal technical review. Unfortunately, these policies and guidelines and strict rules by journals cannot completely eliminate errors and misconduct. Investigators are the determining factor for preventing errors and misconduct in scientific research. To provide meaningful and replicable science to the research community, we suggest that investigators develop laboratory-specific standard operating procedures to ensure transparency, rigor, and reproducibility within the team and outside the team.

As excellently stated by Dr. Nallamothu, the editor-in-chief of Circulation: Cardiovascular Quality and Outcomes, in a recent editorial, “Very few answers in science are clear-cut or simple. Most depend on the critical assumptions we make as part of an iterative process in research. It is time to make those choices clear for others to see, assess, debate, replicate, and build upon” (10). Transparency is the key to build rigor and reproducibility, the key to collaborate in research community, and the key to improve scientific knowledge and discover new molecular mechanisms to advance human health. We expect that the NIH and scientific journals will enhance the present policies and guidelines with ultimate goals to turn scientific discoveries from animal research into preventing and treating human diseases.

## Author Contributions

HL and AD: manuscript design and manuscript editing. HL: manuscript draft. Both authors contributed to the article and approved the submitted version.

## Funding

The authors' research study was supported by National Heart, Lung, and Blood Institute of the National Institutes of Health under Award Numbers R01HL139748 and R35HL155649, and the American Heart Association SFRN in Vascular Disease (18SFRN33900001).

## Author Disclaimer

The content in this commentary is solely the responsibility of the authors and does not necessarily represent the official views of the National Institutes of Health.

## Conflict of Interest

The authors declare that the research was conducted in the absence of any commercial or financial relationships that could be construed as a potential conflict of interest.

## Publisher's Note

All claims expressed in this article are solely those of the authors and do not necessarily represent those of their affiliated organizations, or those of the publisher, the editors and the reviewers. Any product that may be evaluated in this article, or claim that may be made by its manufacturer, is not guaranteed or endorsed by the publisher.
